# Paeonol Protects Against Myocardial Ischemia/Reperfusion-Induced Injury by Mediating Apoptosis and Autophagy Crosstalk

**DOI:** 10.3389/fphar.2020.586498

**Published:** 2021-01-21

**Authors:** Chin-Feng Tsai, Hsing-Hui Su, Ke‐Min Chen, Jiuan-Miaw Liao, Yi-Ting Yao, Yi-Hung Chen, Meilin Wang, Ya-Chun Chu, Yi-Hsin Wang, Shiang-Suo Huang

**Affiliations:** ^1^ Division of Cardiology, Department of Internal Medicine, Chung Shan Medical University Hospital, Taichung, Taiwan; ^2^ School of Medicine, Institute of Medicine, Chung Shan Medical University, Taichung, Taiwan; ^3^ Department of Pharmacology, Chung Shan Medical University, Taichung, Taiwan; ^4^ Department of Parasitology, Chung Shan Medical University, Taichung, Taiwan; ^5^ Department of Physiology, Chung Shan Medical University and Chung Shan Medical University Hospital, Taichung, Taiwan; ^6^ Graduate Institute of Acupuncture Science, China Medical University, Taichung, Taiwan; ^7^ Research Center for Chinese Medicine and Acupuncture, China Medical University, Taichung, Taiwan; ^8^ Department of Photonics and Communication Engineering, Asia University, Taichung, Taiwan; ^9^ Department of Microbiology and Immunology, School of Medicine, Chung-Shan Medical University, Taichung, Taiwan; ^10^ Department of Anesthesiology, Taipei Veterans General Hospital and National Yang-Ming University, Taipei, Taiwan; ^11^ Department of Pharmacy, Chung Shan Medical University Hospital, Taichung, Taiwan

**Keywords:** apoptosis, autophagy, paeonol, myocardial ischemia/reperfusion injury, crosstalk

## Abstract

Many studies have shown that crosstalk exists between apoptosis and autophagy, despite differences in mechanisms between these processes. Paeonol, a major phenolic compound isolated from Moutan *Cortex Radicis*, the root bark of *Paeonia* × *suffruticosa* Andrews (Paeoniaceae), is widely used in traditional Chinese medicine as an antipyretic, analgesic and anti-inflammatory agent. In this study, we investigated the detailed molecular mechanisms of the crosstalk between apoptosis and autophagy underlying the cardioprotective effects of paeonol in rats subjected to myocardial ischemia/reperfusion (I/R) injury. Myocardial I/R injury was induced by occlusion of the left anterior descending coronary artery (LAD) for 1 h followed by 3 h of reperfusion. Paeonol was intravenously administered 15 min before LAD ligation. We found that paeonol significantly improved cardiac function after myocardial I/R injury and significantly decreased myocardial I/R-induced arrhythmia and mortality. Paeonol also significantly decreased myocardial infarction and plasma LDH activity and Troponin-I levels in carotid blood after I/R. Compared with vehicle treatment, paeonol significantly upregulated Bcl-2 protein expression and significantly downregulated the cleaved forms of caspase-8, caspase-9, caspase-3 and PARP protein expression in the I/R injured myocardium. Myocardial I/R-induced autophagy, including the increase of Beclin-1, p62, LC3-I, and LC3-II protein expression in the myocardium was significantly reversed by paeonol treatment. Paeonol also significantly increased the Bcl-2/Bax and Bcl-2/Beclin-1 ratios in the myocardium after I/R injury. The cardioprotective role of paeonol during I/R injury may be due to its mediation of crosstalk between apoptotic and autophagic signaling pathways, which inhibits apoptosis and autophagic cell death.

## Introduction

Recent studies have shown that cardiomyocyte death is the main reason for the poor prognosis of patients with ischemic heart disease (Chiong et al., 2011; Teringova and Tousek, 2017). The most appropriate clinical strategy to limit myocardial infarct size, preserve cardiac function and improve both quality of life and survival in patients with ischemic heart disease is to minimize myocardial ischemia/reperfusion (I/R) injury-induced cardiomyocyte death, such as necrosis, apoptosis, or autophagy (Tian et al., 2013). Necrosis is marked by defective plasma membrane integrity, cellular and organellar swelling, and marked inflammation. Apoptosis and autophagy differ from necrosis, in that neither apoptosis nor autophagy exhibit myocardial inflammation and they share many death signals that regulate cell death, which may be important signaling pathways in myocardial I/R injury (Oerlemans et al., 2013). Apoptosis, characterized by chromatin condensation, nuclear fragmentation, cell shrinkage and production of apoptotic bodies, is a major mechanism of regulated cell death during myocardial I/R injury (Xia et al., 2016). Autophagy is activated in order to remove damaged organelles and stimulate phagocytic clearance of apoptotic cells (Zou et al., 2012). Evidence suggests that autophagy is beneficial during ischemia, functioning as a protein quality controller and cellular homeostasis keeper; however, excessive autophagic activity degrades essential proteins and organelles, leading to progressive consumption of proteins or organelles and autophagic cell death during I/R (Zeng et al., 2016). Even if the mechanisms differ between autophagy and apoptosis, some proteins modulate both autophagy and apoptosis. It is well documented that the crosstalk between autophagy and apoptosis is mediated by the functional and structural interactions between Beclin-1 and Bcl-2. Beclin-1, the mammalian orthologue of yeast Atg6, is part of the class III phosphatidylinositol 3-kinase (PI3K) complex that induces autophagy. Bcl-2 is not only an important regulator in apoptosis, but also an anti-autophagic protein via its inhibitory interaction with Beclin-1 (Pattingre et al., 2005). Notably, after interacting with Bcl-2, Beclin-1 is sequestrated away from the Vps34/class III PI3K complex and autophagy is inhibited, whereas Bcl-2 maintains anti-apoptotic potential (Levine et al., 2008; Ciechomska et al., 2009). Evidence has shown that the interplay between autophagy and apoptosis is closely related to I/R injury, so recent studies have therefore focused on the crosstalk between these cell death processes (Li M. et al., 2016; Dong et al., 2019).

Paeonol, a major phenolic constituent extracted from Moutan *Cortex Radicus*, the root bark of *Paeonia* × *suffruticosa* Andrews (Paeoniaceae), and the root of *Paeonia lactiflora Pall*, is widely used as a nutrient supplement and as an antipyretic, analgesic and anti-inflammatory agent in traditional Chinese medicine. Paeonol is associated with a wide range of biological effects, including antiplatelet aggregation (Koo et al., 2010), antioxidative (Ding et al., 2016) and anti-inflammatory activity (Lei et al., 2016). Paeonol also demonstrates antiatherosclerotic activity via the upregulation of autophagy and inhibition of vascular smooth muscle cell proliferation (Wu et al., 2017), and prevents oxidized low-density lipoprotein (ox-LDL)-induced injury to vascular endothelial cells by inhibiting excessive autophagy, increasing miR-30a levels and reducing Beclin-1 protein expression (Li et al., 2018). Furthermore, paeonol decreases cerebral infarction and neurological deficits in rats subjected to focal cerebral I/R injury (Hsieh et al., 2006; Liao et al., 2016) and has proven cardioprotective effects in rats subjected to myocardial I/R injury, improving hemorrheology indexes, decreasing oxidative injury and repairing endothelial function (Yang et al., 2010). Paeonol has also demonstrated activity against cardiotoxicity induced by epirubicin, a chemotherapeutic agent, by upregulating epirubicin-induced reductions in miR-1 expression and suppressing the phosphatidylinositol 3-kinase (PI3K)/AKT and the mammalian target of rapamycin (mTOR) signaling pathways, and NF-κB signaling pathways (Wu et al., 2018).

None of these previous reports has determined whether the crosstalk between apoptosis and autophagy may play a role in the cardioprotective effect of paeonol. Our investigation is the first to examine the molecular mechanisms involved in the crosstalk between apoptosis and autophagy that underlie the cardioprotective effects of paeonol in rats subjected to myocardial I/R injury.

## Materials and Methods

### Methods

All study protocols were performed in accordance with those in the *Guide for the Care and Use of Laboratory Animals* published by the National Research Council (United States) Committee (National Academies of Sciences, 2011). All efforts were taken to reduce the numbers of sacrificed animals and to minimize animal suffering.

### Animals

All experiments and postoperative animal care were reviewed and approved by the Institutional Animal Care and Use Committee of Chung Shan Medical University (IACUC-1772). We used male Sprague-Dawley rats (LASCO Co., Charles River Technology, Taipei, Taiwan) weighing 250–350 g. The animals were housed in a room under controlled temperature (24 ± 1 °C) and humidity (55 ± 5%) conditions and subjected to a 12:12 h light-dark cycle. They were allowed free access to food and water.

### Surgical Procedure

Myocardial I/R injury was induced by temporary occlusion of the left anterior descending coronary artery (LAD), as per previously described procedures (Wang et al., 2016). Briefly, the rats were anesthetized with a single intraperitoneal injection of urethane (1.25 g/kg) and then placed on a heated, small animal operating table. After tracheotomy, the intubated animals were ventilated with room air through a respirator for small rodents (Model 131, NEMI, United States) with a stroke volume of 15 ml/kg body weight and rate of 60 strokes/min. The jugular vein was cannulated to administer drugs and polyethylene catheters (PE-50) were inserted into the common carotid artery with a Statham P23 XL transducer to continuously monitor heart rate (HR) and arterial blood pressure (BP). A standard 1-lead ECG recorded heart rhythm via silver electrodes attached to the extremities and the adequacy of anesthetic depth. Data were acquired with a MP35 physiological recorder (BIOPAC Systems, Inc., United States).

The heart was exposed via left thoracotomy and the fourth and fifth ribs were sectioned approximately 2 mm to the left of the sternum. The heart was rapidly externalized and inverted. A 6/0 silk ligature was then placed around the left main coronary artery. The heart was repositioned in the chest and the animal was allowed to recover for 15 mins. Animals that developed arrhythmia or sustained a decrease in mean BP to less than 70 mmHg during the procedure were not included in the study. A small plastic snare from a Portex P-270 cannula was threaded through the ligature and placed in contact with the heart. The left coronary artery was then occluded by tightening the ligature and reperfusion was achieved by releasing the tension applied to the ligature (operated groups). Successful ligation of the coronary artery was validated by decreased arterial pressure and ECG changes (increase in R wave and ST segment elevation), which are indicators of ischemia. Sham-operated animals underwent the same surgical procedure, except that the silk ligature that was placed around the left coronary artery was not tied. Changes in HR, BP, and ECG were simultaneously recorded using a personal computer with waveform analysis software (AcqKnowledge, Biopac System, Goleta, CA, United States) before and during the ischemia and reperfusion periods. To evaluate antiarrhythmic effects of paeonol during myocardial I/R injury, we occluded the coronary artery for 1 h and then subjected it to reperfusion for 3 h. The ventricular ectopic activity was evaluated according to the diagnostic criteria advocated by the Lambeth Conventions (Curtis et al., 2013). Incidence and duration of ventricular tachyarrhythmias, including ventricular tachycardia (VT) and ventricular fibrillation (VF), were determined in the surviving rats and in the rats that eventually died. In rats with irreversible VF, the duration of VF was recorded until mean BP was <15 mmHg. To evaluate the effect of paeonol on cardiac function during myocardial I/R injury, a Millar catheter was inserted into the left ventricular cavity via the right common carotid artery and changes in the left ventricular systolic pressure (LVSP), left ventricular diastolic pressure (LVDP), maximal slope of systolic pressure increment (max dP/dt) and diastolic decrement (min dP/dt) were continuously recorded using a Transonic Scisense Pressure Measurement system (SP200, Transonic Scisense Inc., Ontario, Canada).

### Experimental Groups

Most research has shown that the effective dose range for paeonol cardioprotection is 2.5–100 mg/kg by intragastric or intraperitoneal administration (Yang et al., 2010; Li et al., 2012; Ma et al., 2015; Li H. et al., 2016; Wu et al., 2018). However, recent data indicate that paeonol has higher bioavailability by intranasal and intravenous administration routes in rats compared with the intragastric route (Chen et al., 2010; Adki and Kulkarni, 2020). We therefore investigated the cardioprotective effects of 0.1 and 1 mg/kg paeonol by intravenous administration in rats subjected to myocardial I/R injury. In this study, the solution of paeonol (purity ≧ 98%, Cat. sc-205787, Santa Cruz, Dallas, TX, United States) was freshly prepared and intravenously administered 15 mins before LAD ligation. The rats were randomly assigned to one of five groups: 1) Sham-vehicle: rats underwent all surgical procedures except LAD occlusion and were treated with vehicle (0.1% dimethyl sulfoxide [DMSO] in normal saline); 2) Sham-P-1: rats underwent all surgical procedures except LAD occlusion and were treated with paeonol (1 mg/kg); 3) I/R-Control: rats were treated with vehicle and underwent myocardial I/R injury; 4) I/R-P-0.1: rats were treated with paeonol (0.1 mg/kg) and underwent myocardial I/R injury; or 5) I/R-P-1: rats were treated with paeonol (1 mg/kg) and underwent myocardial I/R injury.

### Estimation of Myocardial Infarct Size and Myocardial Damage

The sizes of both the ischemic zone and infarct zone in the rats’ hearts were determined by previously described procedures (Wang et al., 2016). Evaluation of the infarct zone and collection of myocardium samples for further analyses were carried out in rats that survived 1 h of ischemia and 3 h of reperfusion. At the end of the experiment, the coronary artery was reoccluded, and 2.0 ml of 0.1% Evans blue solution was intravenously injected to stain non-ischemic myocardium and determine the area at risk. The heart was then excised and the atria were removed. Ventricular tissues were sliced into 2 mm sections and incubated in tetrazolium dye (2% 2,3,5-triphenyltetrazolium chloride [Sigma, United States] in normal saline) at 37 °C for 30 mins in darkness. The sections were placed in a solution of 10% formaldehyde in saline for 1 day before excision of infarcted (white) tissues. The weight of the infarcted tissues is expressed as a percentage of the total ventricle or the area at risk. In addition, arterial blood collected from the carotid catheter in rats that survived after 1 h of ischemia and 3 h of reperfusion was centrifuged at 3,000 g for 10 mins to isolate plasma and determine *in vivo* heart injury. Myocardial cellular damage was determined using automated clinical analyzers to measure plasma activities of lactate dehydrogenase (LDH) (ADVIA 1800; Siemens Healthcare Diagnostics Inc., NY, United States) and the levels of Troponin-I (Centaur, Siemens Healthcare Diagnostics Inc., NY, United States).

### Western Blot

For reducing the number of animal sacrifices in this study, the effective dose of paeonol 1 mg/kg was applied to investigate the underlying mechanism involved in the cardioprotective effect of paeonol. Protein expression was evaluated by Western blot, as per previously described procedures (Su et al., 2019). The hearts of rats with myocardial I/R injury were homogenized in T-PER Tissue Protein Extraction Reagent (Thermo Scientific, United States) containing protease Inhibitor Cocktail (Thermo Scientific, United States). The homogenates were centrifuged at 10,000 g for 10 mins at 4 °C. Protein concentration was determined with protein assay kits (BioRad, United States) using bovine serum albumin (BSA) as the standard. Samples were mixed with an equal volume of loading buffer (62.5 mM Tris-HCl, pH 6.8, 10% [v/v] glycerol, 2% SDS, 5% [v/v] 2-mercaptoethanol and 0.05% [w/v] bromophenol blue) and heated for 10 min at 95 °C. The mixture was subjected to SDS-PAGE and transferred electrophoretically to nitrocellulose membranes at a constant current of 70 V for 120 mins. Membranes were blocked with 5% (w/v) non-fat milk in PBS containing 0.1% (v/v) Tween 20 (PBST) for 1 h at room temperature. Membranes were reacted with primary antibodies at 37 °C for 1 h. Membranes were washed three times with PBST and HRP-conjugated secondary antibody (1:10,000 dilution) was added and incubated at 37 °C for 1 h to detect primary bound antibody. Reactive proteins were detected by enhanced chemiluminescence (T-Pro Biotechnology, Taiwan R.O.C.) and the density of specific immunoreactive bands was quantified by densitometric scanning.

### Antibodies

#### Primary Antibodies

Anti-caspase 3, anti-caspase 9, anti-caspase 8, anti-PARP (Cell Signaling, Danvers, MA, United States), anti-mTOR (Spring Bioscience, Pleasanton, CA, United States), anti-phospho mTOR (Santa Cruz, Dallas, TX, United States), anti-Bcl-2, anti-Bax (Abcam, Cambridge, MA, United States), anti-p62, anti-Beclin-1, anti-light chain 3 (LC3) and anti-GAPDH (Novus Biologicals, Littleton, CO, United States).

#### Secondary Antibodies

HRP-conjugated goat anti-mouse IgG (H + L) antibody and HRP-conjugated goat anti-rabbit IgG (H + L) antibody (Jackson ImmunoResearch Laboratories, United States).

### Statistical Analysis

All results are presented as the mean ± the standard error of the mean (S.E.M.). Statistical analyses of differences were assessed by analysis of variance (ANOVA) for combined data and followed by the Student-Newman-Keuls test. The difference in the percentage incidence of VT, VF and mortality was analyzed with a χ2 test. *p* < 0.05 was considered to be statistically significant.

## Results

### Hemodynamic Changes

**FIGURE 1:**
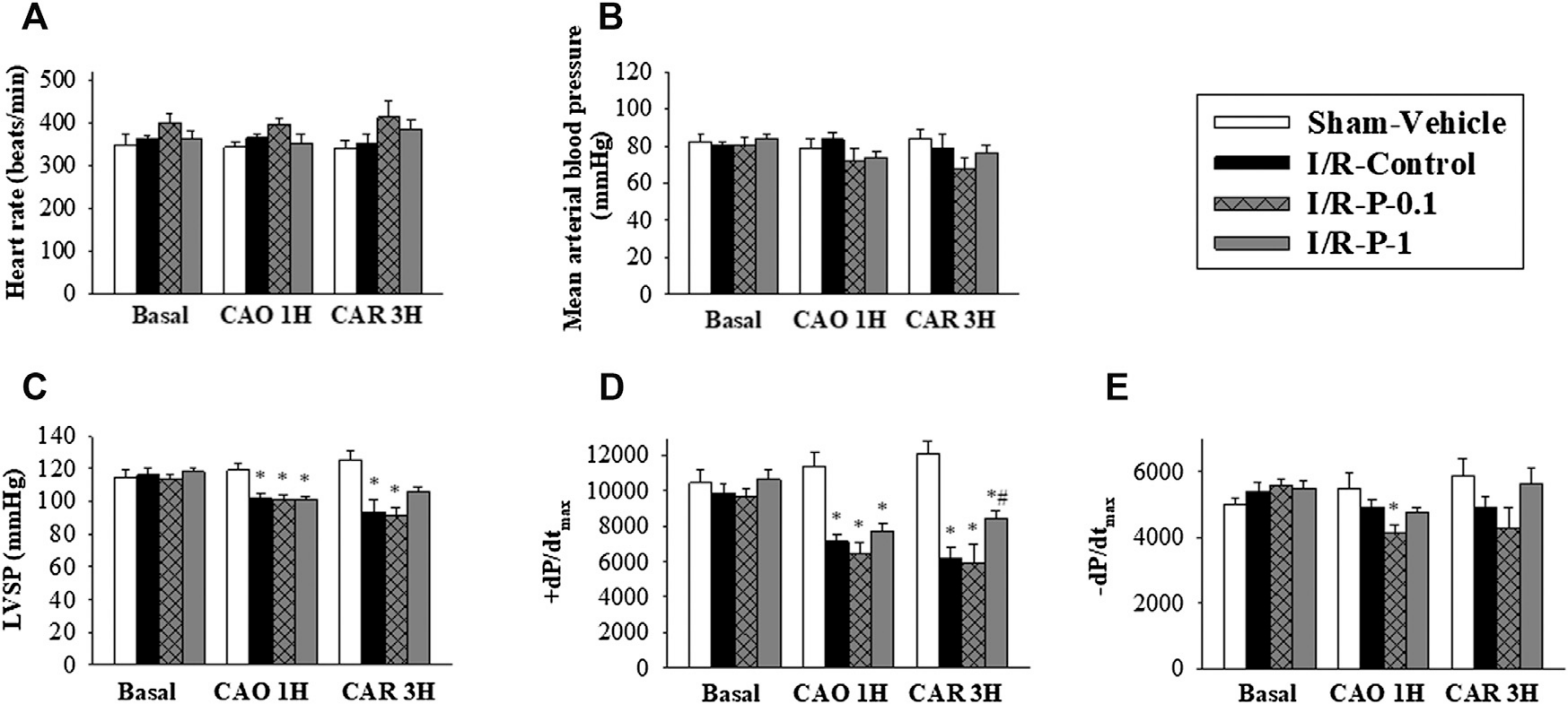
Effect of paeonol on hemodynamic parameters in rats subjected to myocardial I/R injury. **(A)** heart rate, **(B)** mean arterial blood pressure, **(C)** left ventricular systolic pressure (LVSP) **(D)** maximum rates of pressure change in the LV (+dP/dt_max_) and **(E)** minimum rates of pressure change in the LV (−dP/dt_max_) were recorded by physiological parameters during myocardial I-R injury (Basal, pre-ischemic period; CAO1H, 1 h after LAD coronary arterial occlusion; CAR3H, 3 h after LAD coronary arterial reperfusion). Values are means ± S.E.M.; (*n* = 10–11 per group); *p < 0.05 compared with the sham vehicle-treated group; #*p* < 0.05 compared with the I/R-control group.

The hemodynamic parameters did not significantly differ among the four experimental groups in the pre-ischemic period. Intravenous injection of paeonol did not change HR (Figure 1A) or mean BP (Figure 1B) in rats before and during myocardial I/R injury. No significant differences were recorded in LVSP, +dp/dt_max_ or −dp/dt_max_ values before the LAD ligation, amongst the sham, control and paeonol-treated groups. However, the administration of 1 mg/kg paeonol ameliorated the myocardial function of rats, as reflected by the increase in LVSP (Figure 1C) and ±dp/dt_max_ (Figures 1D,E) during myocardial I/R injury and significantly increased +dp/dt_max_ (Figure 1D) after myocardial I/R injury.

### Myocardial I/R Injury-Induced Rhythm Disturbances

The effects of paeonol on arrhythmias and mortality elicited by myocardial I/R injury in anesthetzed rats are shown in Supplementary Table S1. Jugular vein injection of vehicle or paeonol had no induced VT and VF, and none of them died in sham-operated animals. In the control group, severe ventricular arrhythmias occurred at 6–7 mins and peaked at 8–12 mins after LAD ligation. Among the 12 rats in the control group, all animals (100%) exhibited VT (75.44 ± 12.80 s in duration) and 11 rats (92%) exhibited VF (134.36 ± 27.66 s in duration). Administration of paeonol at the dose of 1 mg/kg significantly decreased the incidence of VF (17%), as well as the duration of each (VT: 13.33 ± 6.06 s; VF: 2.00 ± 1.60 s). The mortality rate from myocardial I/R injury was significantly rescued from 50% to 0% in rats treated with paeonol 1 mg/kg.

### Myocardial Infarct Size and Myocardial Damage

**FIGURE 2:**
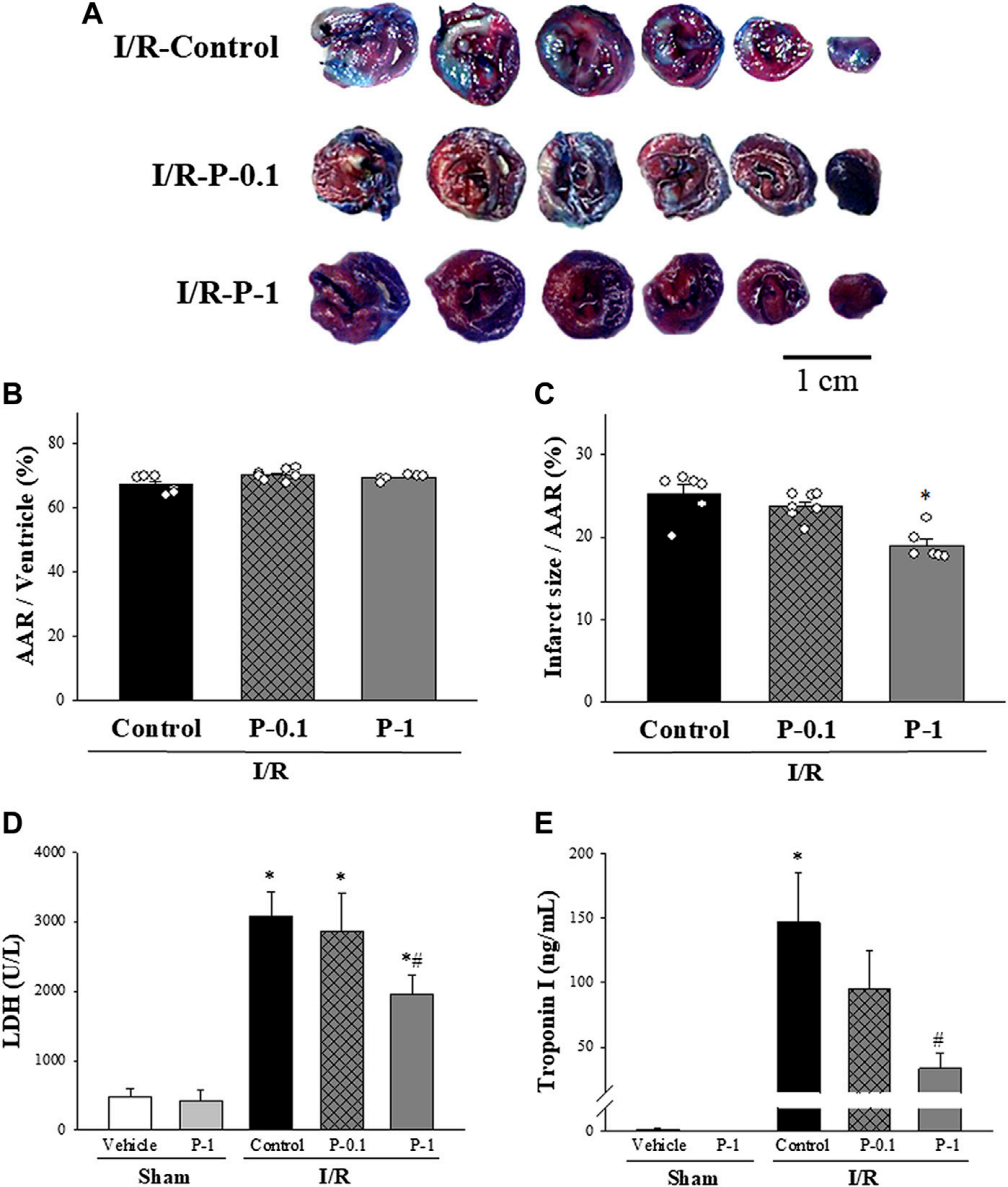
Effect of paeonol on I/R induced myocardial damage in rats. **(A)** Representative Evans blue/TTC staining of heart sections from the control group and different paeonol dose groups (0.1 and 1 mg/kg) after 1 h of ischemia and 3 h of reperfusion. The perfused area is seen in blue; the area at risk (AAR) is seen in red and white. Infarction is seen in white. Quantitative analysis of the AAR/Ventricle % (ratio of non-blue area to total area) (B), and the risk zone infarcted % (ratio of white area to AAR) **(C)** in control and paeonol-treated rats, plasma lactate dehydrogenase (LDH) activity **(D)**, and plasma Troponin-I levels **(E)** in the sham-operated and I/R-operated groups. The results are shown as means ± S.E.M. (*n* = 10–11 per group); *p < 0.05 compared with the sham vehicle-treated group; #*p* < 0.05 compared with the I/R-control group.

The effects of paeonol on myocardial infarct size are shown in Figures 2A–C. There was no significant difference in the AAR between the control and paeonol-treated groups, indicating that in each group, a similar amount of tissue was at risk from myocardial ischemia (Figure 2B). In the control group, the infarct size was 25.20 ± 1.12% of the AAR. Paeonol 1 mg/kg significantly rescued the infarct size to 18.92 ± 0.76% of the AAR (Figure 2C).

Plasma LDH activity and Troponin-I levels were used as diagnostic biomarkers for acute myocardial infarction. The effects of paeonol on LDH activity and Troponin-I levels after myocardial I/R injury are shown in Figures 2D,E. Low LDH activity and Troponin-I levels in the plasma were recorded in the sham-operated animals, whereas both biomarkers were dramatically increased in the control group after myocardial I/R injury. Paeonol 1 mg/kg significantly reduced plasma LDH activity (Figure 2D) and Troponin-I levels (Figure 2E) compared with values in the control group during the same period. These results are consistent with the findings of myocardial infarction.

### Apoptosis and Autophagy Protein Expression

**FIGURE 3:**
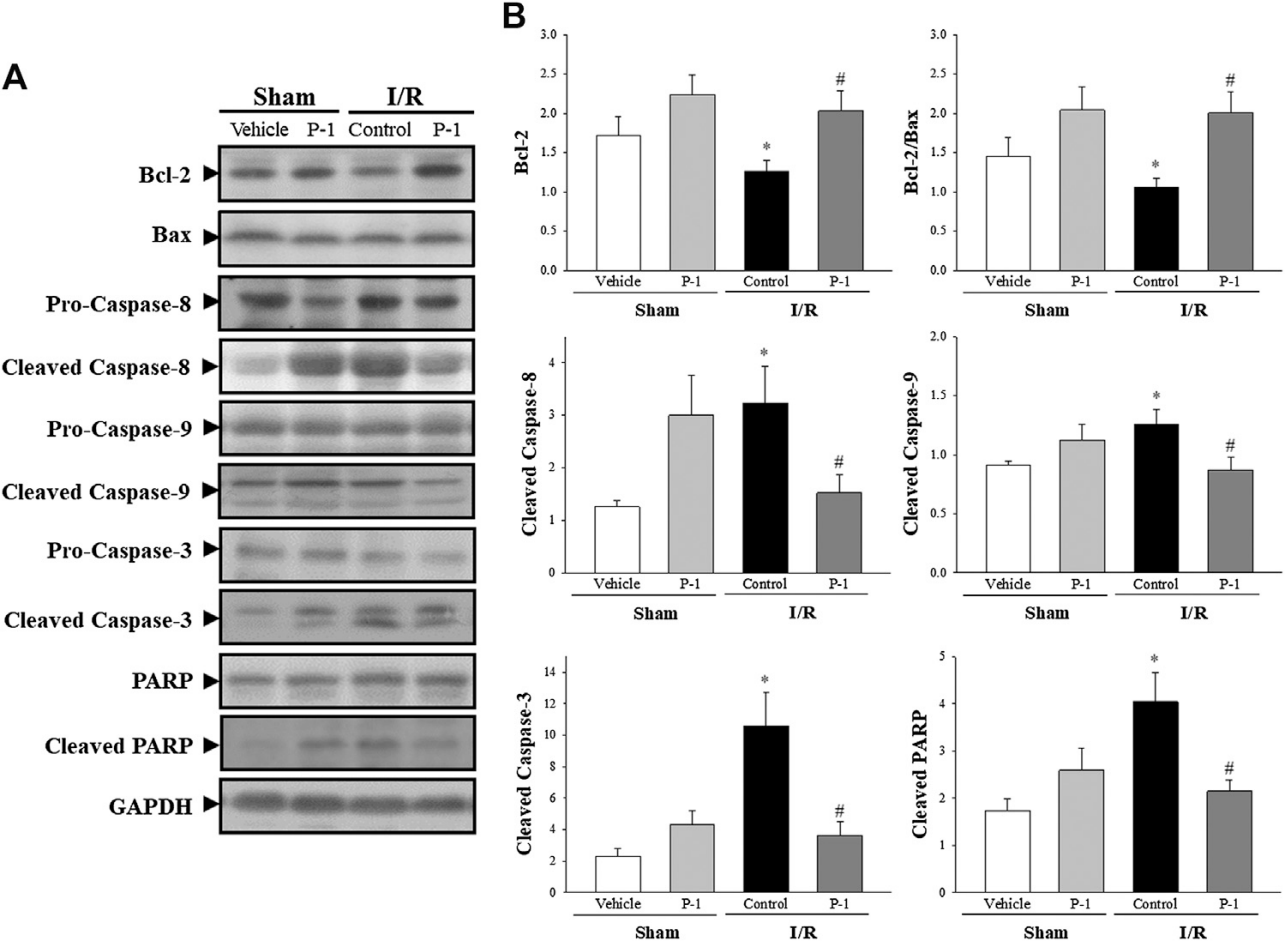
Paeonol decreased apoptosis after myocardial I/R injury**. (A)** Representative images of Western blot results in heart tissue. **(B)** Graphs represent the quantitative differences between vehicle- and paeonol-treated groups in sham- and myocardial I/R-operated animals. GAPDH was used as a loading control for the blots. Data are normalized with the mean expression from the sham vehicle-treated group. *p < 0.05 compared with the sham vehicle-treated group; #*p* < 0.05 compared with the I/R-control group. Values are expressed as means ± S.E.M. (*n* = 6).

Under the sham-operated non-I/R condition, Bcl-2, the cleaved forms of caspase-3, caspase-8, caspase-9 and PARP protein expression in the myocardium did not differ significantly between the groups. Myocardial I/R injury often leads to apoptosis of cardiomyocytes. In this study, we found that myocardial I/R injury significantly reduced the protein expression of Bcl-2 and the ratio of Bcl-2 to Bax, and significantly increased the cleaved forms of caspase-8, caspase-9, caspase-3 and PARP protein expression in the myocardium, compared with the sham-vehicle-treated group. Paeonol treatment significantly reversed changes in protein expression induced by myocardial I/R injury (Figure 3). These results indicate that paeonol attenuates apoptotic cell death in I/R-injured myocardium.

**FIGURE 4:**
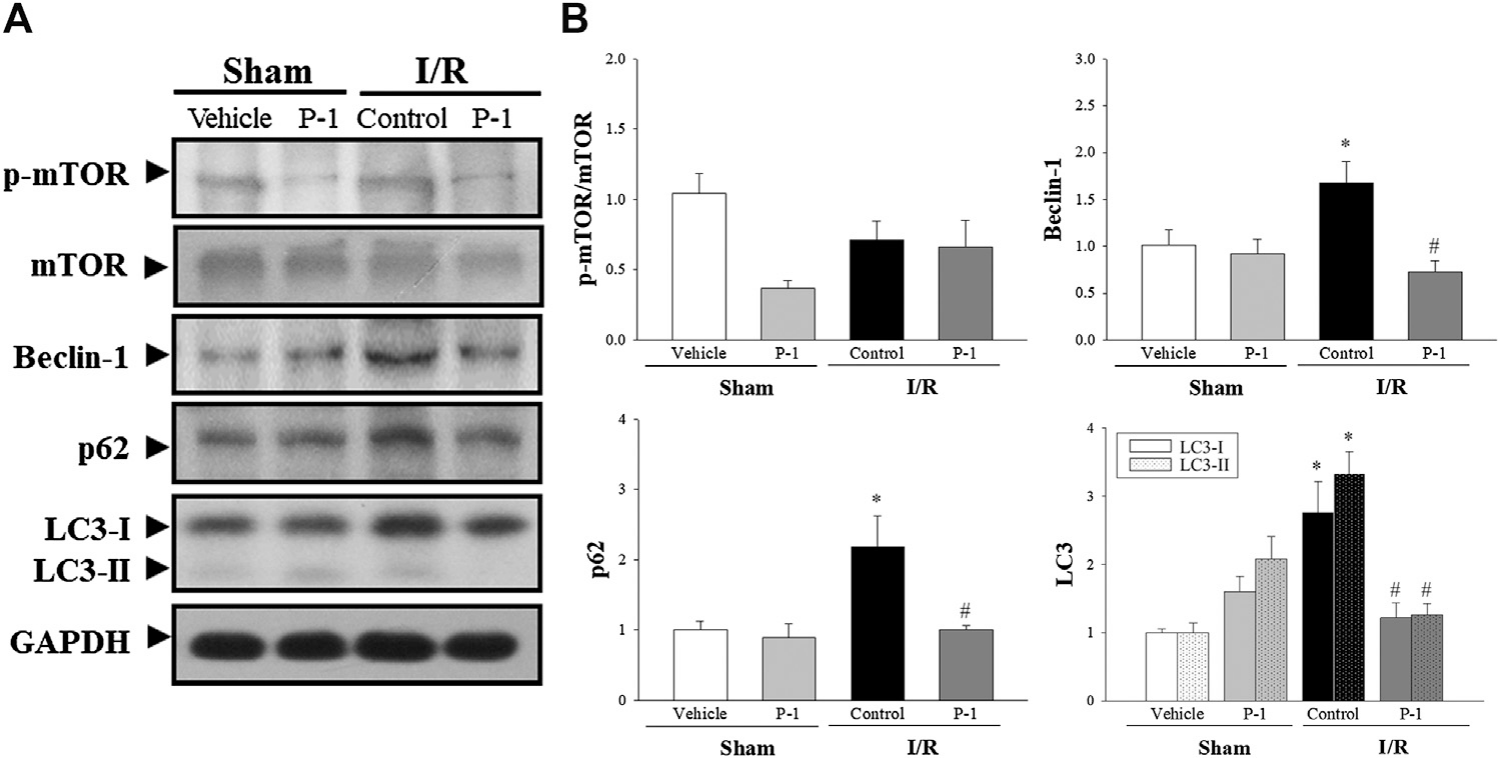
Paeonol decreased autophagy after myocardial I/R injury**. (A)** Representative Western blot images show the expression levels of *p*-mTOR, mTOR, Beclin-1, p62, LC3-I, LC3-II and GAPDH in heart tissue. **(B)** Graphs represent the quantitative differences between vehicle and paeonol-treated groups in sham and myocardial I/R-operated animals. GAPDH was used as a loading control for the blots. Data are normalized with the mean expression from the sham vehicle-treated group. *p < 0.05 compared with the sham-vehicle group; #*p* < 0.05 compared with the I/R-control group. Values are expressed as mean ± S.E.M. (*n* = 6).

We also investigated the role of autophagy in the cardioprotective effects of paeonol in rats subjected to myocardial I/R injury (Figure 4). We found that myocardial I/R injury significantly increased the levels of Beclin-1, p62, LC3-I and LC3-II protein expression in the myocardium in the control group compared with the sham-vehicle-treated group. Paeonol treatment significantly reversed the changes in protein expression induced by myocardial I/R injury. This suggests that paeonol reduced autophagy in the myocardium after I/R injury.

**FIGURE 5:**
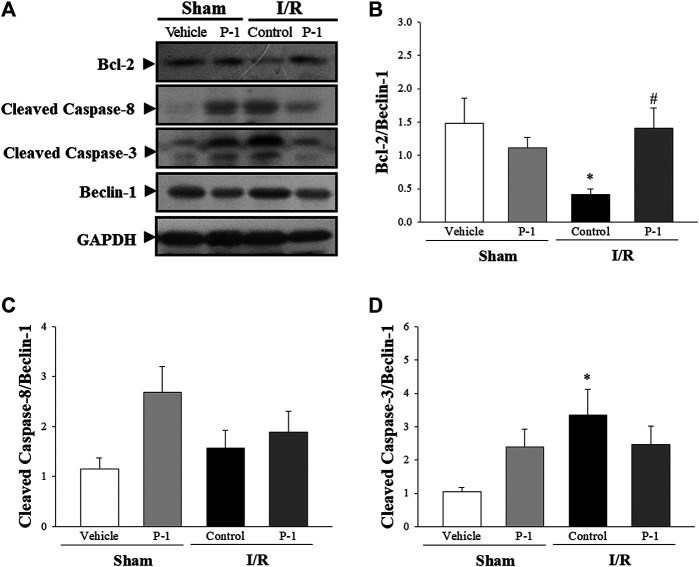
Effect of paeonol on crosstalk between apoptosis and autophagy after myocardial I/R injury in rats**. (A)** Representative images of the Western blots results in heart tissue. Graphs represent the quantitative differences of **(B)** the ratio of Bcl-2 to Beclin-1 **(C)** the ratio of cleaved caspase-8 to Beclin-1 **(D)** the ratio of cleaved caspase-3 to Beclin-1 between vehicle and paeonol-treated groups in sham- and myocardial I/R-operated animals. GAPDH was used as a loading control for the blots. Data are normalized with the mean expression from the sham-vehicle-treated group. *p < 0.05 compared with the sham-vehicle group; #*p* < 0.05 compared with the I/R-control group. Values are expressed as means ± S.E.M. (*n* = 6).

The autophagy induction pathway of Beclin-1 is considered harmful during myocardial I/R injury (Ma et al., 2015). In this study, we found that myocardial I/R injury significantly decreased the ratio of Bcl-2 to Beclin-1 protein expression and significantly increased the ratio of cleaved form of caspase-3 to Beclin-1 protein expression in the control group compared with the sham-vehicle-treated group. Paeonol treatment reversed the protein expression ratio changes induced by myocardial I/R injury. We found that paeonol significantly increased the ratio of Bcl-2 to Beclin-1 protein expression, but without changing the ratio of cleaved-caspase 8 and cleaved-caspase 3 to Beclin-1 protein expression after myocardial I/R injury compared with the control group (Figure 5). Finally, paeonol significantly decreased the expression of Beclin-1 protein in the myocardium in the paeonol-treated group compared with the control group.

## Discussion

In this study, we provide evidence showing that paeonol protects the heart against myocardial I/R injury by improving cardiac function, reducing myocardial extrinsic and intrinsic apoptosis and attenuating autophagy.

Paeonol is a major phenolic compound of Moutan *Cortex Radicis* that is widely used as a nutrient supplement and in traditional Chinese medicine to regulate human disorders by removing blood stasis, promoting blood flow, attenuating inflammatory responses, relieving pain and eliminating heat (Fu et al., 2012). Yang et al. reported that paeonol improves blood hemorrheology, reduces oxidative injury and repairs endothelial function, and acts as an effective cardioprotective agent in a rabbit model of myocardial ischemia induced by intravenous administration of pituitrin (Yang et al., 2010). Our results suggest that administration of paeonol 1 mg/kg significantly suppressed the incidence of VF, shortened the durations of VT and VF, and completely prevented mortality during myocardial I/R injury. In addition, pretreatment with paeonol significantly reduced cardiac infarction size and protected against cardiomyocyte injury, as indicated by the decreasing carotid blood Troponin-I levels and LDH activity, which serve as indicators of cellular damage, in rats subjected to cardiac ischemia for 1 h and reperfusion for 3 h. Our results indicate that paeonol is a potent cardioprotective agent in rats with myocardial I/R injury.

Among the many underlying mechanisms of myocardial I/R injury, apoptosis is one of the most important (Xia et al., 2016). Apoptosis is a form of programmed cell death that is positively and negatively regulated by the Bcl-2 family, which includes both pro- and antiapoptotic proteins. The ratio of proapoptotic to antiapoptotic molecules helps determine the susceptibility of cells to a death signal (Gross et al., 1999). In this study, we found that compared with vehicle treatment, paeonol significantly increased the ratio of Bcl-2 to Bax in the myocardium after ischemia for 1 h and reperfusion for 3 h. Much evidence suggests that suppressing apoptosis may protect the heart against myocardial I/R injury (Wang et al., 2015; Wu et al., 2015). We found that paeonol treatment significantly reduced the protein expression levels of cleaved forms of caspase-8, caspase-9, caspase 3 and PARP, when compared with vehicle treatment. These results illustrate that paeonol upregulates Bcl-2 levels and suppresses apoptosis to protect the heart from I/R injury in rats through inhibition of both intrinsic and extrinsic apoptotic pathways.

Besides apoptosis, autophagy may also contribute to myocardial I/R injury (Matsui et al., 2007). Evidence indicates that autophagy plays dual roles in myocardial I/R injury; at low levels, autophagy is protective, by degrading damaged mitochondria and protein aggregates, whereas excessive autophagy such as during myocardial I/R may be deleterious for cardiac function, due to overwhelming degradation of essential proteins and organelles (Li M. et al., 2016; Wang et al., 2017). It is known that Beclin-1-dependent autophagy mediates autophagic cell death during myocardial I/R injury (Peng et al., 2013). Beclin-1 is a key autophagic protein that plays a vital role in autophagy in myocardial I/R and therefore serves as a marker for autophagy. Elevated Beclin-1 protein expression induces high levels of autophagy, while inhibition of Beclin-1 protein expression correlates with decreased autophagy and reduced cardiac myocyte death induced by myocardial I/R injury (Matsui et al., 2007). In our studies, we found that paeonol significantly decreased Beclin-1 expression in the occluded zone of rats subjected to myocardial I/R injury. In addition to inhibiting Beclin-1 expression, paeonol also attenuated myocardial I/R injury by dramatically reducing p62, LC3-I and LC3-II expression. Both LC3 and p62 are routinely used as biomarkers to monitor autophagic levels. During autophagy initiation, cytosolic-associated protein LC3-I is converted to the membrane-bound LC3-II form and then binds to the adaptor protein p62, facilitating the autophagic degradation of ubiquitinated protein aggregates in autolysosomes (Aghaei et al., 2019). Our results suggest that paeonol decreases autophagic cell death during myocardial I/R injury.

In this study, we focused on investigating the underlying mechanisms through which the crosstalk between apoptosis and autophagy was involved in the cardioprotective effect of paeonol on rats subjected to myocardial I/R injury. Both apoptosis and autophagy are two highly regulated biological processes with complex protein networks that play a vital role in tissue homeostasis, development, and disease. Autophagy is inhibited downstream of apoptosis induction due to Beclin-1 cleavage by caspases, most prominently caspase-3 (Fairlie et al., 2020). Recent evidence indicates that active caspase-8, a death receptor effector, can be degraded by autophagy, suggesting the existence of a feedback mechanism that cross-regulates autophagy and apoptosis (Hou et al., 2010). However, we found that paeonol did not affect the ratios of cleaved-caspase 8 and cleaved-caspase 3 to Beclin-1 protein expression after myocardial I/R injury compared with those ratios in the control group. It also appears that several inducers of apoptosis, such as p53 and Bcl-2, can activate autophagy. Phosphorylation of p53 is known to inhibit the activity of mTOR through AMPK activation (Mendieta et al., 2019). In this study, we found that paeonol treatment did not change the protein expression of mTOR in rats after myocardial I/R injury, which means that p53 phosphorylation is not involved in the cardioprotective effects of paeonol. Mitochondria-associated proteins are responsible for interactions between autophagy and apoptosis. Bcl-2 is an anti-apoptotic protein that interferes with Beclin-1 at the mitochondrial outer membrane, resulting in dual regulation of apoptosis and autophagy. In this study, we found that paeonol (1 mM) significantly increased cell viability against OGD/R insult in H9c2 cells, and the protective effect is reversed by Bcl-2-selective inhibitor ABT199 (Supplementary Figure S2). The specific Bcl-2 inhibitor, ABT199, is a selective small-molecule B-cell lymphoma 2 Homology 3 (BH3) mimetic, has been shown to disrupt the BH3 dependent Bcl-2/Beclin-1 interaction, thereby induce apoptosis and autophagy (Chiang et al., 2018; Folkerts et al., 2019). Therefore, we suggest that the cardioprotective effects of paeonol against I/R injury may mediated by Bcl-2/Beclin-1 interaction. It is generally believed that Beclin-1-mediated autophagy is not only regulated by its own expression, but is also promoted by the binding of Beclin-1 and Bcl-2 (Marquez and Xu, 2012). Previous studies have shown that the balance between Bcl-2 and Beclin-1 protein expression affects the levels of autophagy (Yi et al., 2020). We therefore explored the association between Bcl-2 and Beclin-1 protein expression in the crosstalk between apoptosis and autophagy in rat heart after I/R injury. Our data verified that compared with vehicle treatment, treatment with paeonol significantly upregulates the expression of Bcl-2 and downregulates the expression of Beclin-1, increasing the ratio of Bcl-2 to Beclin-1 in the I/R-injured myocardium. Upregulation of Bcl-2 and downregulation of Bax, Beclin-1, LC3, and p62 following paeonol treatment suggests that crosstalk exists between apoptosis and autophagy processes during myocardial I/R. Thus, the cardioprotective effects of paeonol against I/R injury may involve upregulation of Bcl-2 that inhibits apoptosis and prevents autophagic cell death.

## Conclusion

**FIGURE 6:**
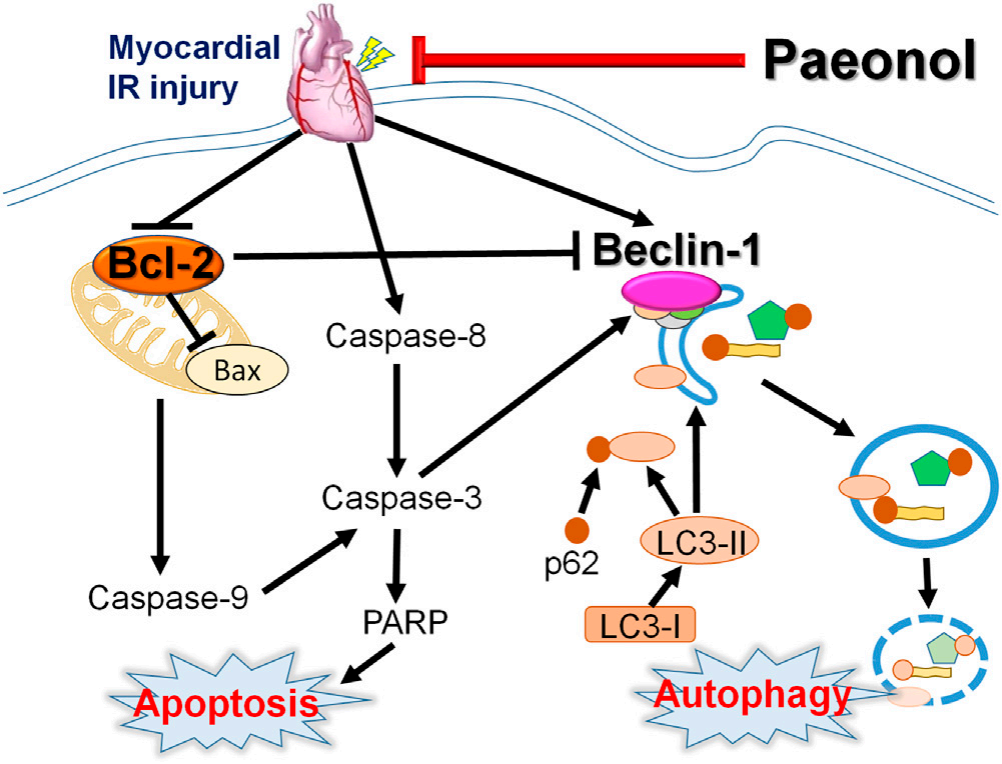
Schematic diagram of paeonol mediating apoptosis and autophagy crosstalk in myocardial I/R injury.

Our results provide valuable insights into the signaling pathways involved in programmed cell death, including crosstalk between apoptosis and autophagy, in paeonol-induced amelioration of myocardial I/R injury in rats (Figure 6). Paeonol shows potential as a preventive treatment for ischemic heart diseases or post-coronary revascularization.

## Data Availability Statement

The raw data supporting the conclusions of this article will be made available by the authors, without undue reservation, to any qualified researcher.

## Ethics Statement

The animal study was reviewed and approved by The Institutional Animal Care and Use Committee of Chung Shan Medical University.

## Author Contributions

C-FT and S-SH designed research and provided funding. K-MC, H-HS, J-ML, Y-TY, Y-HW, Y-HC, MW, Y-CC, and S-SH completed animal experiments, conducted molecular biology experiments and analyzed data. C-FT, Y-CC, Y-HW, and S-SH wrote the paper. All authors read and approved the final manuscript.

## Funding

This work was supported financially by research grants from Chung Shan Medical University Hospital (CSH-2017-C-021 and CSH-2018-C-031), and the Ministry of Science and Technology (MOST 107-2320-B-040-025) of Taiwan to C-FT and S-SH.

## Conflict of Interest

The authors declare that the research was conducted in the absence of any commercial or financial relationships that could be construed as a potential conflict of interest.

## Abbreviations

±dp/dtmax, the maximal rate of left ventricular pressure increase and decrease; AAR, Area at risk; Bax, B-cell lymphoma-2-associated X protein; Bcl-2, B-cell lymphoma-2; BP, arterial blood pressure; HR, heart rate; I/R, ischemia/reperfusion; LAD, left anterior descending coronary artery; LC3, light chain 3; LDH, lactate dehydrogenase; LVSP, left ventricular systolic pressure; mTOR, mammalian target of rapamycin; PARP, poly (ADP-ribose) polymerase; VT, ventricular tachycardia; VF, ventricular fibrillation.
