# Supplementary material for: Paeonol Protects Against Myocardial Ischemia/Reperfusion-Induced Injury by Mediating Apoptosis and Autophagy Crosstalk
Source: Front Pharmacol. 2021 Jan 21;11:586498. doi: 10.3389/fphar.2020.586498 (PMC7858273; doi:10.3389/fphar.2020.586498)
Supplement: Supplementary file 1 [file table1.docx]

**Table**

Effect of Paeonol on Arrhythmias Induced by Myocardial I/R Injury in Anesthetized Rats.

| n | | | | **VT** | | | | | **VF** | | | | **Mortality** | |
| --- | --- | --- | --- | --- | --- | --- | --- | --- | --- | --- | --- | --- | --- | --- |
|  |  | | | Incidence (%) | | Duration (sec) | | Incidence (%) | | | Duration (sec) | |  | |
| **Sham**  Vehicle | | 7 | **-** | | - | | - | | | - | | 0 | |  |
| P-1 | | 4 | **-** | | - | | - | | | - | | 0 | |  |
| **I/R**  Control | | 12 | 100 | | 75.44 ± 12.80 | | 92 | | | 134.36 ± 27.66 | | 50 | |  |
| P-0.1 | | 16 | 94 | | 17.70 ± 4.73* | | 56 | | | 47.94 ±12.97* | | 44 | |  |
| P-1 | | 12 | 66 | | 13.33 ± 6.06* | | 17* | | | 2.00 ± 1.60* | | 0* | |  |

VT: ventricular tachycardia; VF: ventricular fibrillation.

Values for duration of VT and VF are shown as the mean ± S.E.M. *Statistical difference at the level of *p* < 0.05 as compared with I/R-Control.
