# Supplementary material for: Paeonol Protects Against Myocardial Ischemia/Reperfusion-Induced Injury by Mediating Apoptosis and Autophagy Crosstalk
Source: Front Pharmacol. 2021 Jan 21;11:586498. doi: 10.3389/fphar.2020.586498 (PMC7858273; doi:10.3389/fphar.2020.586498)
Supplement: Supplementary file 2 [file table2.docx]

**Cell culture**

The H9c2 embryonic rat heart-derived (ventricular) cells (myoblasts) was purchased from AddexBio (cat no. C00031002) and cultured in Dulbecco's modified Eagle's medium (DMEM) supplemented with 10% fetal bovine serum (FBS), 1% penicillin (10,000 units/mL) and streptomycin (10,000 µg/mL) both from GIBCO (Grand Island, NY, USA). Cells were maintained in a humidified incubator consisting of 5% CO_2_ and 95% air at 37°C.

**Oxygen Glucose Deprivation/Reoxygenation (OGD/R) Model**

In this study, we used oxygen and glucose deprivation followed by reoxygenation (OGD/R) in H9c2 cells to mimic the *in vitro* model of myocardial I/R injury. H9c2 cells were seeded onto 96-well plates at a density of 1×10^4^ per well for 24 hours. The medium was replaced and the cells were exposed to the different treatment: Control (0.1% DMSO), paeonol (0.1, 1 mM) (Thabassum Akhtar Iqbal et al., 2020), with or without Bcl-2-selective inhibitor (ABT199, 30 nM) (Diaz-Flores et al., 2019) for 1 hr before OGD. To simulate anoxia in H9c2 cells, plated cells were exposed to a glucose-free and serum-free solution and incubated for 16 hours in direct heat CO_2_/multi-gas incubator (astec, japan) saturated with 95% N_2_ and 5% CO_2_ at 37°C. Then, the cells were incubated with normal culture medium in CO_2_ incubator (NAPCO) saturated with 95% air and 5% CO_2_ at 37°C (normoxic conditions) for 2 hours as reoxygenation (Testai et al., 2015).

**Cell viability assay**

After OGD/R, cell viability was assessed using the Cell Counting Kit-8 (CCK8; targetmol, USA). H9C2 cells were seeded in 96-well plate (1×10^4^ cells/ml) and underwent OGD/R, then the cells were incubated with 10% CCK-8 solution for 1 hr at 37 °C under normoxic conditions, and the absorbance (ODs) value at 450 nm was detected by TECAN Sunrise ELISA Reader to calculate cell viability. Cell viability were expressed as percentage of the absorbance of control-treated cells exposed to normoxic conditions.

Diaz-Flores, E., Comeaux, E.Q., Kim, K.L., Melnik, E., Beckman, K., Davis, K.L., Wu, K., Akutagawa, J., Bridges, O., Marino, R., Wohlfeil, M., Braun, B.S., Mullighan, C.G., and Loh, M.L. (2019). Bcl-2 Is a Therapeutic Target for Hypodiploid B-Lineage Acute Lymphoblastic Leukemia. *Cancer Res* 79**,** 2339-2351.

Testai, L., Barrese, V., Soldovieri, M.V., Ambrosino, P., Martelli, A., Vinciguerra, I., Miceli, F., Greenwood, I.A., Curtis, M.J., Breschi, M.C., Sisalli, M.J., Scorziello, A., Canduela, M.J., Grandes, P., Calderone, V., and Taglialatela, M. (2015). Expression and function of Kv7.4 channels in rat cardiac mitochondria: possible targets for cardioprotection. *Cardiovascular Research* 110**,** 40-50.

Thabassum Akhtar Iqbal, S., Tirupathi Pichiah, P.B., Raja, S., and Arunachalam, S. (2020). Paeonol Reverses Adriamycin Induced Cardiac Pathological Remodeling through Notch1 Signaling Reactivation in H9c2 Cells and Adult Zebrafish Heart. *Chemical Research in Toxicology* 33**,** 312-323.
