# Supplementary figures and images for: Paeonol Protects Against Myocardial Ischemia/Reperfusion-Induced Injury by Mediating Apoptosis and Autophagy Crosstalk
Source: Front Pharmacol. 2021 Jan 21;11:586498. doi: 10.3389/fphar.2020.586498 (PMC7858273; doi:10.3389/fphar.2020.586498)

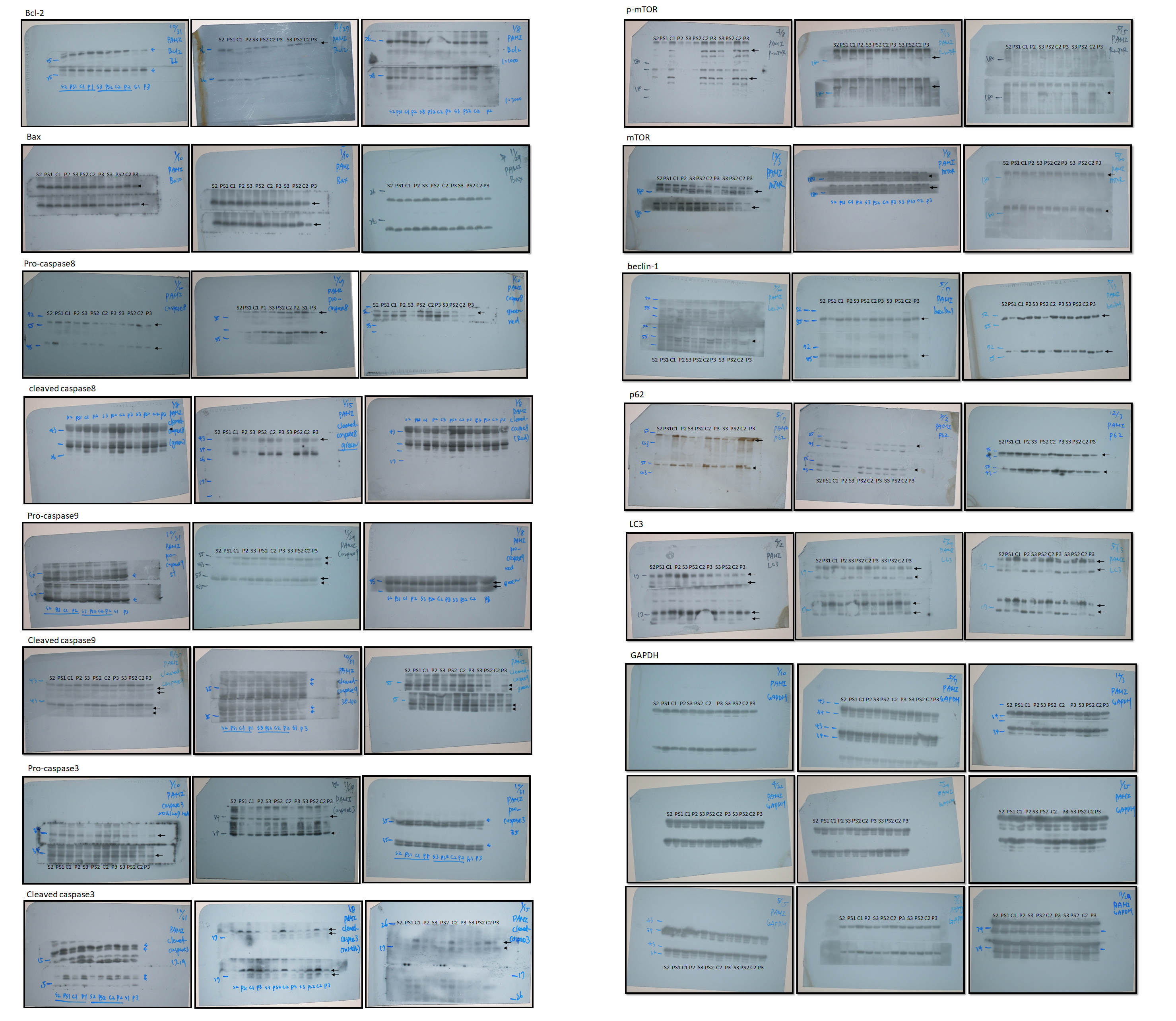

Supplement: Supplementary file 3 [file image1.tif]

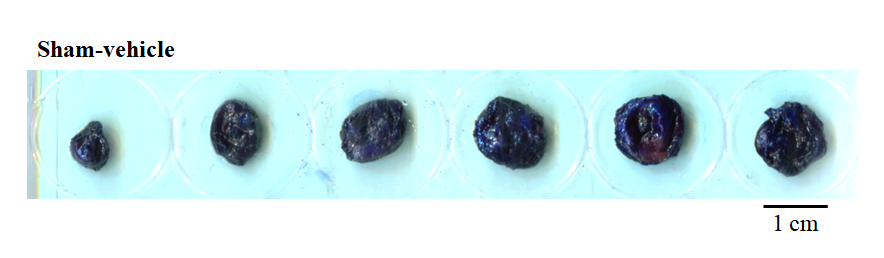

Supplement: Supplementary file 4 [file image2.tif]

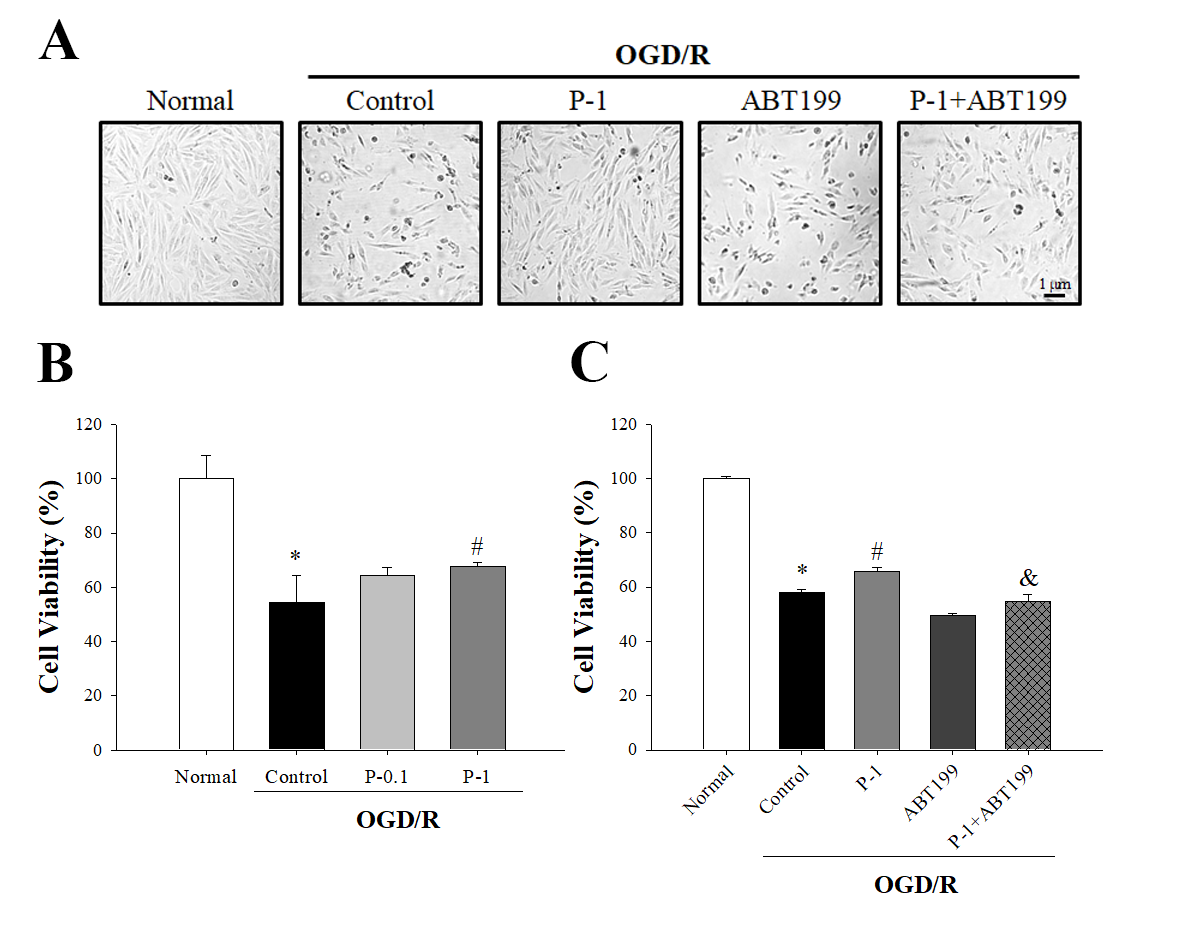

Supplement: Supplementary file 5 [file image3.tif]
